# Retrieval of Brain Tumors with Region-Specific Bag-of-Visual-Words Representations in Contrast-Enhanced MRI Images

**DOI:** 10.1155/2012/280538

**Published:** 2012-11-25

**Authors:** Meiyan Huang, Wei Yang, Mei Yu, Zhentai Lu, Qianjin Feng, Wufan Chen

**Affiliations:** School of Biomedical Engineering, Southern Medical University, Guangzhou 510515, China

## Abstract

A content-based image retrieval (CBIR) system is proposed for the retrieval of T1-weighted contrast-enhanced MRI (CE-MRI) images of brain tumors. In this CBIR system, spatial information in the bag-of-visual-words model and domain knowledge on the brain tumor images are considered for the representation of brain tumor images. A similarity metric is learned through a distance metric learning algorithm to reduce the gap between the visual features and the semantic concepts in an image. The learned similarity metric is then used to measure the similarity between two images and then retrieve the most similar images in the dataset when a query image is submitted to the CBIR system. The retrieval performance of the proposed method is evaluated on a brain CE-MRI dataset with three types of brain tumors (i.e., meningioma, glioma, and pituitary tumor). The experimental results demonstrate that the mean average precision values of the proposed method range from 90.4% to 91.5% for different views (transverse, coronal, and sagittal) with an average value of 91.0%.

## 1. Introduction

Digital images in the medical field, such as visible, ultrasound, X-ray, CT, MRI, and nuclear images help radiologists to make a diagnosis. However, searching for images with the same anatomic regions or similar-appearing lesions according to their visual image features in a huge image dataset is a challenging task. To address this problem, possible and promising solution to indexing images with minimal human intervention is presented, the content-based image retrieval (CBIR) system [1]. In the medical field, the CBIR system commonly contains two types of application: retrieval of the same anatomical regions [2–4] and retrieval of similar lesions [5–8]. This study concentrates on the retrieval of similar brain tumors in MRI images although the proposed methods can be applicable to other organs with lesions. MRI is usually selected to delineate soft tissue, especially when attempting to diagnose brain tumors [9]. Hence, the brain MRI image of a patient with a tumor can help radiologists to obtain useful information on the tumor category. In this study, we focus on three types of brain tumors in T1-weighted contrast-enhanced MRI (CE-MRI) images, namely, meningiomas, gliomas, and pituitary tumors, because these three have higher incidence rates than other brain tumors in clinics. The goal of the proposed CBIR system is to assist radiologists in making a diagnostic decision by sending a query image (tumor) to a retrieval system. The most relevant images (tumors), which are visually similar and fall into the same pathological category as the query image (tumor), are then returned as diagnostic aids.

**Figure 1:**
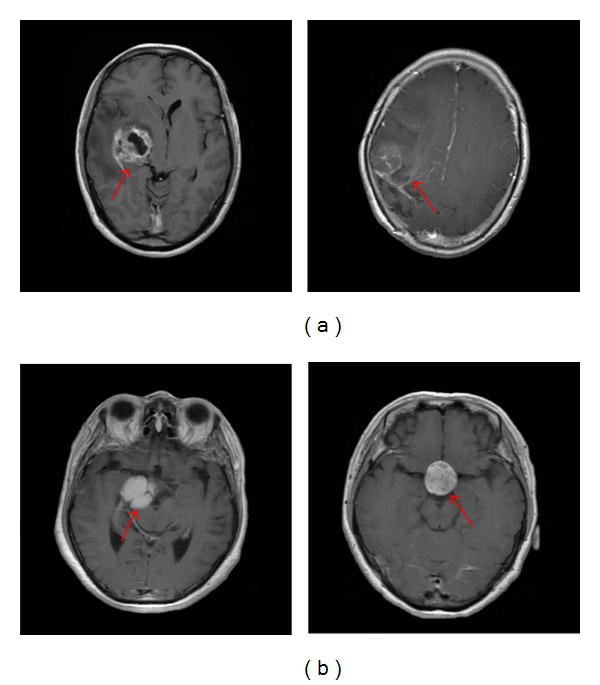
Two examples of brain tumors with varying appearances in T1-weighted CE-MRI images. The red arrows in each image are used to indicate the tumors. (a) Two gliomas in different patients have dissimilar appearances. (b) A meningioma (left) and a pituitary tumor (right) in different patients have similar appearances.

Generally, the category retrieval for brain tumors is a difficult task due to the complex appearances of tumors. For example, same-type brain tumors in different patients may present different appearances, or different-type brain tumors may show visual similarity (Figure 1). Therefore, additional distinctive features integrating domain knowledge need to be presented to construct the CBIR system. One contribution of this current work is to introduce the region-specific bag-of-visual-words (BoW) model, which can incorporate spatial information into the BoW model and integrate domain knowledge to improve the performance of the proposed CBIR system.

Another challenge in the development of the CBIR system is how to define the relevance between images. If the visual features are directly used to compute the image relevance, the performance of the CBIR system may decrease because the low-level image features cannot always capture the semantic concepts in the images. Hence, the distance metric learning algorithms are used to learn an optimal similarity metric to overcome the gap between the high-level semantic concepts and the low-level visual features in images. As of this writing, numerous distance metric learning algorithms have been introduced to learn an optimal similarity metric [10–12]. Among these algorithms, close-form metric learning (CFML) can be solved in a closed form instead of an iterative process, which simplifies computations. Therefore, CFML is used in this paper.

The rest of the paper is organized as follows.  Section 2 provides a brief review of related work.  Section 3 presents the details of the proposed CBIR system.  Section 4 gives the experimental studies.  Section 5 discusses the results and the ideas for future work.

## 2. Related Work

### 2.1. CBIR Systems for Brain Tumors

Numerous studies have focused on constructing a CBIR system for brain tumors in MRI images [13–15]. In [14], regions of interests are generated as queries to retrieve the relevant brain MRI images of pediatric patients. Moreover, Dube et al. [15] proposed a method for the image retrieval of glioblastoma multiforme (GBM) and non-GBM tumors on MRI images. In the proposed method, regions containing lesions were also manually segmented. Using the same operation as that used in these studies, brain tumors in the MRI images are manually outlined to form the dataset of the CBIR system in this paper. However, the category retrieval of brain tumors is performed on the T1-weighted CE-MRI images from different patients and different views (transverse, coronal, and sagittal), as opposed to other MRI modalities used in the previous studies.

### 2.2. Feature Extraction for Image Representation

To compare the relevance between the query image and the images in the dataset, feature extraction for image representation is conducted in the CBIR system. The comparison comes down to comparing the features of the images. Thus, feature extraction for image representation is a crucial factor in developing a CBIR system. To date, the formulation of a proper image representation remains a common problem in classification and retrieval system design. In previous studies, researchers introduced shape [16, 17], intensity, and texture information [18–23] to extract informative features for image representation. Geometric cues such as edges, contours, and joints extracted from an image can be used to extract powerful shape descriptors when objects in the image can be clearly separated from the background or surroundings. Unfortunately, in brain tumor images, the tumors boundaries are usually unclear. Hence, shape information cannot offer a satisfactory delineation of the tumor regions. In the medical domain, ascertaining the intensity and texture features is becoming increasingly important, because these can implicitly reflect the tissue categories and the fine details contained within the tissues in an image. Several methods are commonly used to describe the intensity and texture features of brain MRI images, including gray level cooccurrence matrix (GLCM) [19, 23], discrete wavelet transformation (DWT) [18, 20, 22], and Gabor filters [21]. GLCM can capture the spatial correlation among adjacent pixels. However, the optimal interpixel distance in a specific situation is an inherent problem to be addressed using the GLCM method. DWT can achieve simultaneous feature localization in time and frequency domains, and its performance is highly dependent on the wavelet basis and the number of decomposition levels. Gabor filters with various scales and rotations can be used to form a filter bank, which is appropriate for texture representation and discrimination [21, 24]. However, Gaussian smoothing in the filter bank can result in blurring, meaning that fine local details in the images can be lost.

Unlike the commonly used texture extraction methods mentioned, a BoW model [25–27] can be successfully applied to image classification and CBIR systems. The BoW model first learns a vocabulary of visual words with a number of image patches. Then, an image representation is built to capture the signature distribution of these words. The BoW method can also provide a way to design task-specific image representation. Hence, it can be adopted to build a suitable image representation for tumor retrieval in brain MRI images. However, the basic BoW model ignores all information about the spatial layout of the patches, which is extremely important in image representation. In response to this problem, researchers added spatial information to the BoW model for general scene and object recognition tasks. For instance, Savarese et al. [28] proposed shape information to model the spatial correlations with visual words. In addition, Lazebnik et al. [29] introduced a spatial pyramid method by partitioning the image into increasingly finer spatial subregions, and combining all the BoW histograms in each subregion as the final image representation to incorporate global and local information. In spite of the power of these kinds of approaches, they are not all suitable for medical images due to the complex and different intensity distributions in medical images. Therefore, the importance of the BoW model with spatial information is gradually emerging in medical tasks. In [30], spatial coordinates were added to the feature vector to obtain spatial information for X-ray image retrieval. Moreover, the spatial geometry was constructed using the co-occurrence matrix to deal with the visual words that differentiate neoplastic from benign tissues in endomicroscopic images [31]. The approaches proposed in these studies have achieved effective performance for medical image classification and retrieval. However, the retrieval of brain tumors in MRI images remains a challenging task due to varieties associated with tumor location, shape, and size properties (Figure 1). Thus, for the characteristics of brain tumors in MRI images, a novel extension of the orderless BoW model, called the region-specific BoW, is presented in this paper.

## 3. Materials and Methods

### 3.1. Image Data

In this paper, the proposed CBIR system is based on two-dimensional (2D) slices because in Chinese clinical practice; the acquired and available brain CE-MRI images are 2-D slices with a large slice gap. Therefore, the construction of a CBIR system based on 2-D slices for clinical applications is practical.

**Figure 2:**
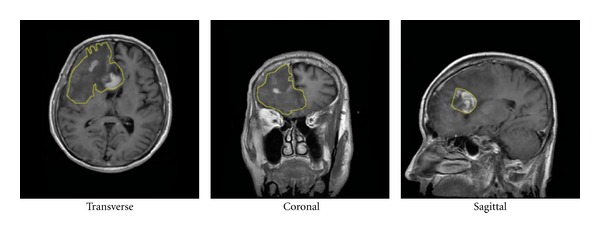
Different views of gliomas in a patient.

The T1-weighted brain CE-MRI dataset used in this study was acquired at Nanfang Hospital, Guangzhou, China, and General Hospital, Tianjin Medical University, China, from 2005 to 2010 using spin-echo-weighted images with a 512 × 512 matrix. The dataset contains the information of 233 patients with brain tumors, wherein three types of brain tumors are apparent, namely, meningiomas, gliomas, and pituitary tumors. For each patient, the radiologists first consulted the patient pathology report to obtain the pathology type and then labeled the images. Next, the radiologists selected one to fourteen slices that best represented the pathology from each patient's full set of image volume. Thus, all tumor images with different views (transverse, coronal, and sagittal) were also presented in the dataset. Considering different views individually in the experiments is practical because the appearance of a tumor in a patient varies depending on the different views (Figure 2). For each view in different patients, one slice was randomly chosen to comprise the dataset of the view.  Table 1 lists the details of image data used in the experiments. In addition, to extract the region-specific features, all tumors in the images were manually outlined by three experienced radiologists who dealt with all of the images independently. Afterward, the radiologists discussed together and reached a consensus regarding the segmentation of every tumor in each image.

### 3.2. Overview of the CBIR System

**Figure 3:**
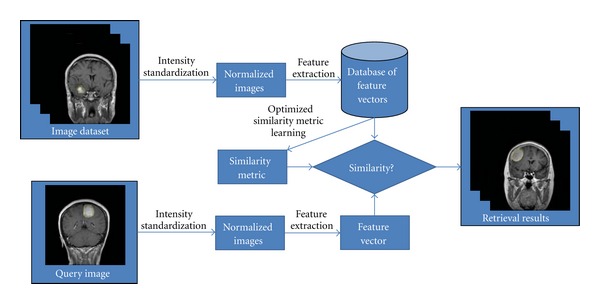
Flowchart of the proposed CBIR system.

In this paper, the construction of the CBIR system for brain tumors in CE-MRI images is finalized through the following steps: intensity standardization of brain CE-MRI images, feature extraction of manually outlined brain tumors in the normalized CE-MRI images, acquisition of the optimized similarity metric using the distance metric learning algorithm, and finally, retrieval of the relevant images for the query image. The flowchart of the proposed CBIR system is illustrated in Figure 3. The techniques employed to perform these processing steps are also explained in detail below.

### 3.3. Image Intensity Standardization

MRI is a useful tool to describe the brain tissue. However, its main limitation is that the intensity values for the same tissue in different MRI images fall into a wide range, which can significantly affect the performance of numerous image-processing techniques in MRI images. Thus, an intensity normalization method that standardizes the intensity values in MRI images is presented in this paper for subsequent feature extraction and brain tumor retrieval. Nyul et al. [32] proposed a two-step method consisting of a training step and a transformation step. With this processing, intensities in the transformed images have consistent tissue meanings. Therefore, the two-step normalization method is employed as the preprocessing method for the MRI images in this paper. For simplicity, the intensity standardization method that uses three histogram landmarks [32] is chosen. The three landmarks are intensities corresponding to the lower, 50th, and upper percentiles of the foreground of the scene [32].

### 3.4. Region-Specific BoW Representation

After the intensity standardization, features are extracted from the normalized MRI images by using the proposed region-specific BoW method. In this paper, the basic BoW model is first described. Afterwards, the region-specific BoW model that incorporates spatial information into the original BoW model is presented.

#### 3.4.1. BoW Model

The BoW model is summarized in four steps. First, patches represented by local descriptors are sampled in each image of the given image dataset. Second, a visual vocabulary is constructed by a clustering algorithm, and each of the cluster centers is a visual word. Third, the visual vocabulary obtained ahead can be used to quantize the local descriptors of a new image. Finally, a BoW histogram is constructed to represent an image by recording the frequency of each visual word in the image.

In the BoW model, the choices of the patch sampling method and the local descriptor are two basic tasks that affect the type of visual words generated. Common patch sampling methods include the densely sampling scheme, random selection, or the interested point detector. The patches are also simultaneously represented using local descriptors. Two local descriptors are often used: scale-invariant feature transform (SIFT) descriptor [33] and raw patch [34].

The next step of the BoW model is to cluster a subset of the patches to build a visual vocabulary. The *k*-means clustering algorithm, which attempts to minimize the distance between *k* clusters and the training data, is applied to locate visual words because of its simplicity and effectiveness.

To represent an image, a set of the patches sampled from every pixel in the image is mapped to a new feature vector of *k* features, where *k* is the number of *k*-means centroids. In this paper, hard-assignment coding is utilized as the encoder for feature mapping. Given the visual words *w* in a vocabulary, an image representation in the BoW model is defined as follows:

(1) $$\begin{array}{c} x\left(w_{i}\right)=\frac{1}{n}\sum_{c=1}^{n}\{\begin{array}{cc} 1 & \text{if}\;i=\underset{j}{\arg\;\min}{||w_{j}-p_{c}||}^{2}\\ 0 & \text{otherwise}, \end{array} \end{array}$$

where *n* is the number of patches in an image and *p*_*c*_ is the patch *c*. Then, an image representation in the BoW model is built and treated as a “bag” filled with visual words.

As mentioned in Section 2, the BoW model loses spatial information among patches due to the orderless collection of visual words into a “bag.” Therefore, we mainly focus on a novel extension of the orderless BoW model, which indicates the location of patch extraction and adds spatial information to the BoW model.

#### 3.4.2. Construction of the Region-Specific BoW Model

**Figure 4:**
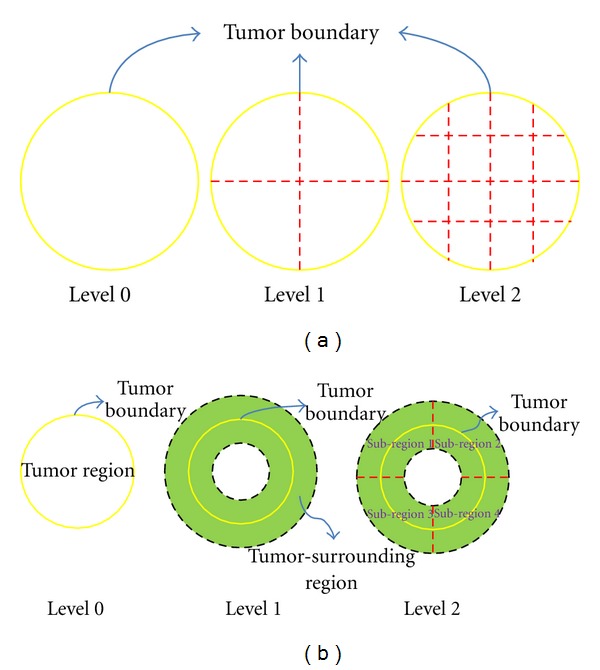
Dividing a tumor image in different modes. To ensure impartiality in the comparison between these two modes, we implement the partition in the tumor region instead of in the whole image as in [29]. (a) The partition mode used in [29]. There are 21 regions in this mode. (b) The partition mode used in our paper. There are six regions: the tumor region, the tumor-surrounding region, and the four subregions.

The proposed BoW model with spatial information was inspired by the idea of region partition [29]. In [29], a spatial pyramid BoW method was introduced by dividing the image into increasingly finer spatial subregions and combining all the BoW histograms in each subregion as the final image representation.  The region partition mode on a tumor with three levels in [29] is shown in Figure 4(a). However, the previously mentioned partition method [29] may be not the best choice for the brain tumor images due to the complex and different intensity distributions in brain tumor images. Therefore, domain knowledge about the brain tumor images is considered in this paper.

Brain tumors commonly do not have a fixed shape or size, even for the same tumor category. However, the intensity values of the same tumor category may fall into a narrow range in normalized brain tumor images. Additionally, contrast enhancement in brain tumor CE-MRI images makes the intensities more discriminative in identifying different tumor categories. On the other hand, the same tumor category is often located in similar places in the abnormal human brain. For instance, meningiomas are usually next to the skull, gray matter, and cerebrospinal fluid. Pituitary tumors are adjacent to sphenoidal sinus, optic chiasma, and internal carotid arteries. Gliomas typically involve white matter and are surrounded by edema. Thus, intensities in the brain tumor and category information of the tumor-surrounding tissues are two important clues in identifying different tumor categories in brain images. Based on this idea, a brain tumor in an MRI image is separated into two major regions: tumor region and tumor-surrounding region (level 0 and level 1 in Figure 4(b), resp.).

The region-specific BoW model based on these two separated tumor regions in the brain images is then presented.

**Figure 5:**
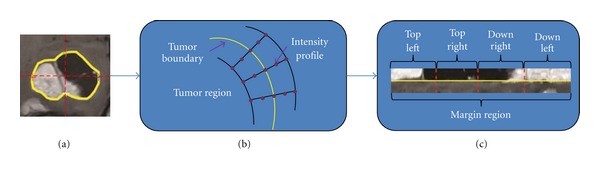
Flow diagram of the intensity profile and the margin region extraction. (a) An outlined tumor. (b) The extraction of the intensity profile in the tumor. In this figure, there are five sampling pixels on each intensity profile. (c) The margin region and its four subregions of the tumor.

First, intensity profiles are employed to capture the intensity variation of the tumor-surrounding region. Each intensity profile is a vector of image intensity values, including the intensities of some pixels along the tumor boundary normal. These pixels are sampled from within the tumor to outside of the tumor (see Figure 5(b)). The extraction of the intensity profile is given below. The Gaussian kernel is used to smoothen the points on the tumor boundary and to prevent the points on the tumor boundary from being disturbed by the noise, which can change the direction of the boundary normal. The Gaussian kernel is defined in one dimensionality as

(2) $$\begin{array}{c} G_{1D}\left(X;\sigma \right)=\frac{1}{\sqrt{2\pi }\sigma }e^{-X^{2}/2\sigma^{2}}, \end{array}$$

where **σ** is the standard deviation. Here, the first derivative of *G*_1*D*_(*X*; *σ*) is used to convolve with the points on the tumor boundary. Let *b*(*x*, *y*) be the coordinate of all points on the tumor boundary in an image. The coordinate of points after convolution is given by

(3) $$\begin{array}{c} B\left(x^{\prime },y^{\prime }\right)=b\left(x,y\right)*G_{1D}{\left(X;\sigma \right)}^{\prime }. \end{array}$$

The angles of the boundary normal are computed using

(4) $$\begin{array}{c} \theta =\arctan\left(\frac{y^{\prime }}{x^{\prime }}\right). \end{array}$$

With the angle *θ*_*i*_, the coordinates of all the points corresponding to an intensity profile *i* are denoted by

(5) $$\begin{array}{c} X_{i}=x_{i}+l\times \cos\theta_{i},\\ Y_{i}=y_{i}+l\times \sin\theta_{i}, \end{array}$$

where *l* is the distance between the points along the tumor boundary normal and the point on the boundary. However, the coordinates (*X*_*i*_, *Y*_*i*_) may not exactly be in a pixel on the image. Thus, linear interpolation is adopted to locate the corresponding pixels in the image. Finally, the intensity values associated with the pixels are extracted to form the intensity profile. Using the extraction of all intensity profiles around a tumor, a rectangle called the margin region is constructed. In this margin region, the length of the intensity profile refers to the width, and the number of points on the tumor boundary is its length. The extractions of the intensity profile and the margin region are shown in Figures 5(b) and 5(c), respectively.

The choices of the patch sampling method and the local descriptor are two basic tasks in constructing the BoW model. Therefore, these two factors must be considered first. The interest point detector [35] is a popular sampling method. However, unlike natural scene images, brain MRI images have less meaningful interest points. Hence, the sparse distinctive points cannot adequately represent an MRI image. For this reason, all information in the region of interest (tumor region or margin region) is used by densely sampling the patches. On the other hand, raw patch is used as the local descriptor because intensity is an important clue in the application of image retrieval for brain MRI. In this paper, a raw patch is a rectangular patch of fixed size around a pixel with the corresponding intensities in an image. Each raw patch is then transformed to a one-dimensional feature vector to simplify the subsequent computation. A popular alternative descriptor to the raw patch is the SIFT descriptor, which is invariant to the scale and rotation. Several studies also show that SIFT is advantageous in the scenery images [25, 36]. The retrieval performance on different local descriptors is given in the experiments to define the system parameter set.

Before constructing the visual vocabulary, a preprocessing stage is applied to the sampled raw patches. Normalization and whitening [37, 38] are two common methods for data preprocessing. Given a vector-represented patch *p*, *p* is normalized by *P* = (*p* − *u*)/*σ*, where *u* and *σ* are the mean and the standard deviations of the *p* vector, respectively. The normalized vector *P* is then linearly transformed to a new vector $\tilde{P}$, which is white. In other words, the components in vector $\tilde{P}$ are uncorrelated, and their variances equal unity. In detail, let *C *be the covariance matrix of *P*; thus, *C* can be defined by

(6) $$\begin{array}{c} C=E\left\{PP^{T}\right\}. \end{array}$$

Here, the eigenvalue decomposition of the covariance matrix *C* is used for whitening as follows:

(7) $$\begin{array}{c} C=VDV^{T}, \end{array}$$

where *V* and *D* are the orthogonal matrix of eigenvectors and the corresponding eigenvalues of *C*, respectively. Now, the whitening transformation of vector *P* can be conducted by

(8) $$\begin{array}{c} \tilde{P}=VD^{-1/2}V^{T}P. \end{array}$$

The preprocessing method used in this study can enhance the local contrast and augment the information in the patch data.

**Figure 6:**
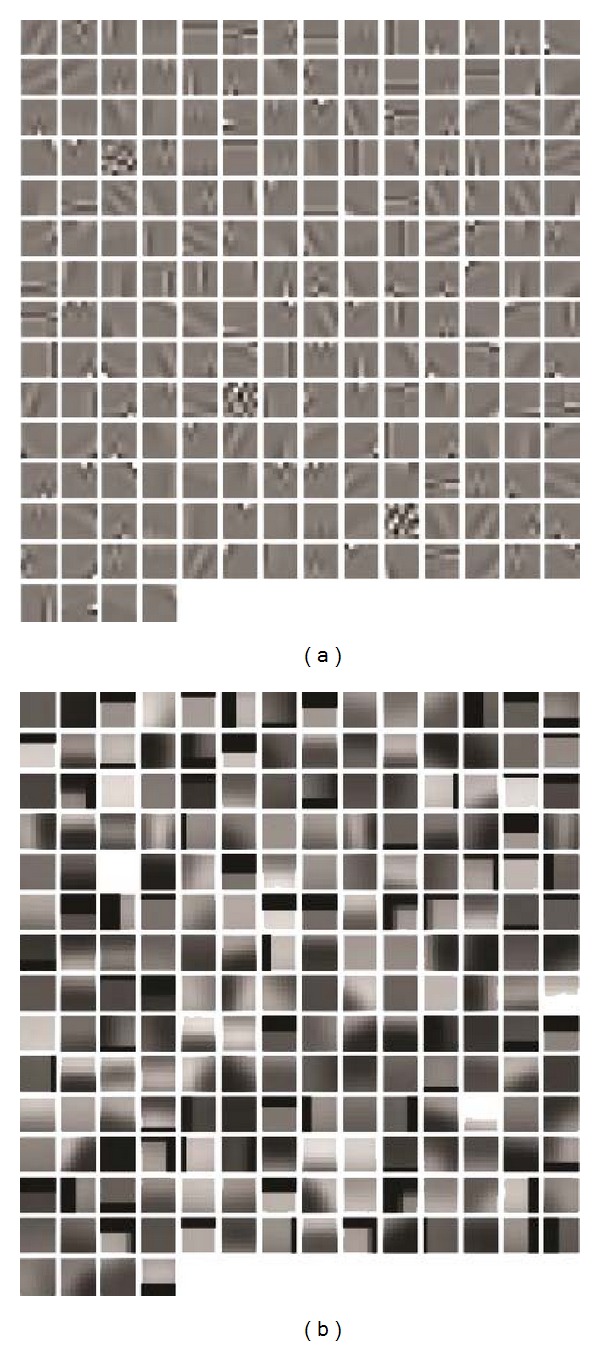
Visual vocabularies constructed in the margin region with a patch size of 7 × 7 and a vocabulary size of 200. (a) Patches with normalization and whitening. (b) Patches without normalization and whitening.

Subsequently, two visual vocabularies are built using the corresponding preprocessed patches sampled within the tumor region and the tumor margin region, respectively. The difference between the current proposed scheme and the basic BoW model mentioned is that only the vector-represented patches densely sampled from the tumor region and the margin region, respectively, are used rather than every pixel in an image to create the vocabularies. This process makes the created vocabularies more region-specific. In other words, given the same vocabulary size, image representation constructed by region-specific vocabulary is more representative than that constructed by a universal vocabulary using all of the information in the image. An example of the region-specific vocabulary is shown in Figure 6.

**Figure 7:**
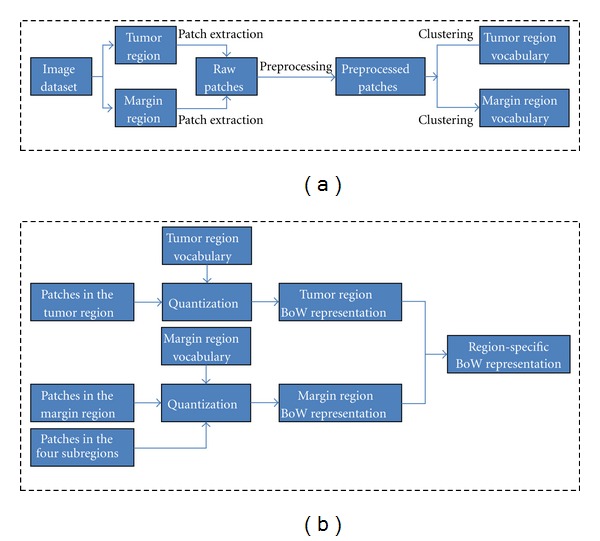
Flow diagram of the region-specific BoW representation construction. (a) The construction of tumor region and margin region vocabularies. (b) The construction of the region-specific BoW representation in a given image. The patches in (b) are obtained via the same procedures as shown in (a).

A given image can now be represented by mapping the preprocessed patches to the corresponding generated vocabularies of words. However, to add more spatial information for the patch extraction, the margin region is separated into four finer subregions based on the four directions of a tumor (i.e., top left, top right, down right, and down left), as seen in Figures 4(b) and 5(c). Therefore, total of six regions in three levels are presented in the proposed method (see Figure 4(b)). To build the image representation in the margin region, the patches sampled in the margin region and the four subregions are mapped to the vocabulary in the margin region, respectively. Then, the BoW histograms for each region are combined as the margin region BoW representation. If the number of words is *k*_1_ in the margin region vocabulary, then the margin region BoW representation is a vector with 5∗*k*_1_ dimensionalities. Thus, the image is now represented by two BoW histograms; one is the tumor region BoW representation, and the other one is the tumor margin region BoW representation. Finally, these two BoW histograms are concatenated to form the proposed region-specific BoW representation as the image representation for the tumor on a brain image. The construction of the region-specific BoW representation is shown in Figure 7.

The proposed feature extraction scheme offers some advantages. First, the preprocessing method for patches can make the components uncorrelated and enhance local contrast within a patch data. This attribute can bring discriminative information to the BoW model. Second, the intensity profile introduced in this paper implicitly suggests the category information of the tumor-surrounding tissues. Moreover, the sampling locations of pixels on a profile give weak spatial distribution information on the tumors in the brain. Treating all intensity profiles around a tumor as a rectangle, which is inherently rotation-invariant to the tumor, can also ignore the directions of patch extraction. Third, the proposed region-specific BoW model can incorporate spatial information into the BoW model and capture important characteristics associated with the brain tumors in MRI images.

### 3.5. Distance Metric Learning

In the image retrieval phase, the similarity between the feature vectors of the query image and the images in the dataset is measured. However, the extracted features may not be directly linked to the tumor category. In other words, a gap exists between the high-level semantic concepts and the low-level visual features in images. If we use common distance metrics such as the Euclidean distance or *χ* ^2^ distance to measure the similarity of the features, the CBIR system cannot perform well. As these distance metrics ignore the labeled data in the training set, they may bring statistical regularity to improve the retrieval performance. To tackle this problem, a distance metric learning algorithm for automatically learning a distance metric with labeled data in the image dataset is presented. Moreover, well-designed distance metrics can perform better than the Euclidean distance on the CBIR system [39, 40]. Thus, the distance metric learning algorithm is used to embed semantic information to the region-specific BoW representation in this paper.

Let *x*_*i*_ and *x*_*j*_ be the D-dimensional feature vectors of two different images. The squared Mahalanobis distance between feature vectors *x*_*i*_ and *x*_*j*_ is

(9) dM(xi,xj)=||L(xi−xj)||22=(xi−xj)TLTL(xi−xj)=(xi−xj)TM(xi−xj),

where *L* is a transformation matrix with *d* × *D*, *M* = *L* ^*T*^ *L*, and *M* is a positive semidefinite matrix. Distance metric learning aims to find a linear transformation matrix *L* to project the image features to a new feature space, making the squared Mahalanobis distance between data with the same labels closer and that with different labels farther.

Numerous researchers have proposed distance metric learning algorithms to find an optimal projection *L* or a metric *M* for minimizing an objective function. Among the various kinds of distance metric learning algorithms, CFML is a simple and effective algorithm that can achieve a closed-form solution. In the dataset, the pathological categories of tumors are known. Tumors in the same category share the same label and vice versa. The feature vectors of images with the same label are defined as similar, whereas those with different labels are defined as dissimilar. Let *S* and *D* be the set of similar pairs and the set of dissimilar pairs, respectively, defined by

(10) $$\begin{array}{c} S:\left(x_{i},x_{j}\right)\in S\;\text{if}\;x_{i},x_{j}\;\text{are}\;\text{similar},\\ D:\left(x_{i},x_{j}\right)\in D\;\text{if}\;x_{i},x_{j}\;\text{are}\;\text{dissimilar}. \end{array}$$

The optimal transformation matrix L* of CFML is defined as follows:

(11) $$\begin{array}{c} f\left(L^{*}\right)=\underset{L}{\arg\;\min}\mathrm{tr}\left(L^{T}\left(M_{S}-M_{D}\right)L\right), \end{array}$$

(12) $$\begin{array}{c} \text{s}.\text{t}.\;L^{T}M_{S}L=I, \end{array}$$

where tr⁡(·) denotes the matrix trace and

(13) $$\begin{array}{c} M_{S}=\frac{1}{\left|S\right|}\sum_{\left(x_{i},x_{j}\in S\right)}\left(x_{i}-x_{j}\right){\left(x_{i}-x_{j}\right)}^{T},\\ M_{D}=\frac{1}{\left|D\right|}\sum_{\left(x_{i},x_{j}\in D\right)}\left(x_{i}-x_{j}\right){\left(x_{i}-x_{j}\right)}^{T}. \end{array}$$

The solution of the optimal transformation matrix L* is given by the matrix of eigenvectors associated to the largest eigenvalues of the matrix *M*_*S*_^−1^ *M*_*D*_. With the optimal transformation matrix, the squared Mahalanobis distance between two feature vectors in two different images can be computed. Here, CFML tries to minimize the squared Mahalanobis distance between similar pairs while simultaneously maximizing the squared Mahalanobis distance between dissimilar pairs.

In this paper, the number of samples is small while the number of features is large. In other words, there is a small-sample large-feature problem even when *M*_*S*_ is nonsingular. To overcome this problem, a regularization form of CFML is introduced by replacing *L* ^*T*^ *M*_*S*_ *L* = *I* with *L* ^*T*^(*M*_*S*_ + *λI*) *L* = *I*, where *λ* (*λ* > 0) is a regularization parameter. The regularization parameter *λ* is experimentally tuned on a cross-validation set (see Section 4).

### 3.6. Retrieval Evaluation Measures

In this section, the retrieval evaluation measures for the proposed CBIR system are presented. First, let *N* be the number of images in the dataset and let *R*_*j*_ be the relevance of an image *j* for a given query image, wherein *R*_*j*_ ∈ {0,1} (1 for relevant if *j* and the query image belong to the same class, and 0 otherwise). Second, when a query image is presented to the CBIR system, the system ranks the images in the dataset by the increasing order of the squared Mahalanobis distance, that is, from the most similar to the least similar. Then, for a given number of samples retrieved, the precision *P*_*K*_ and recall *R*_*K*_ are computed:

(14) $$\begin{array}{c} \text{Precision}=\frac{\sum_{j=1}^{K}R_{j}}{K},\;\;\text{Recall}=\frac{\sum_{j=1}^{K}R_{j}}{\sum_{j=1}^{N}R_{j}}, \end{array}$$

where *K* = 1,…, *N* is the number of samples retrieved. Simultaneously, the precision-recall pairs for varying numbers of retrieved samples are usually plotted in a precision-recall curve to evaluate the retrieval system. Third, precision at the top *K* retrieved samples (Prec*@K* in short), which only considers the topmost results returned by the system. This measure is denoted by

(15) $$\begin{array}{c} \text{Prec}@K=\frac{1}{K}\sum_{j=1}^{N}R_{j}1\left\{\pi \left(x_{j}\right)\leq K\right\}, \end{array}$$

where *π*(*x*_*j*_) represents the position or rank of the retrieved image *x*_*j*_ in the ranked sequence, and 1{·} is the indicator function. Fourth, average precision (AP) is defined by

(16) $$\begin{array}{c} \text{AP}=\frac{1}{\sum_{j=1}^{N}R_{j}}\sum_{j=1}^{N}R_{j}\times \text{Prec}@j. \end{array}$$

Finally, the mean of AP over all the queries is called the mean average precision (mAP), which is mainly used in our experiments to evaluate the overall retrieval performance.

## 4. Experiments and Results

The tuning of the system parameters is a fundamental component in using the BoW model in a retrieval task. Therefore, we use the T1-weighted brain CE-MRI dataset to tune several parameters of the proposed CBIR system: the choice of local descriptors, the size of patches and vocabularies, and the parameters of CFML. Then, we show comparative results of the category retrieval of brain tumors with different feature extraction methods in the T1-weighted brain CE-MRI dataset.

### 4.1. Experimental Settings

In the following experiments, category retrievals of brain tumors in different views were conducted individually, and fivefold cross-validation was used to evaluate the retrieval performance. All experiments were repeated five times, and the final results were reported as the mean and standard deviations of the results from the individual runs. For each run, 152 images were used for training and 38 images were used for testing in the transverse view. The separation scheme in the coronal view was similar in the transverse view due to the use of the same number of images in the two views (see Table 1). In the sagittal view, 172 images were used for training and 43 images were used for testing. At the same time, there was no joint set between the training and test datasets in all of the experiments. For the given training and test datasets, each image in the test dataset was adopted as a query to retrieve the training dataset to report performance.

The pixels on each intensity profile were extracted from inside the tumor to outside the tumor to form the margin region, with each side including 15 pixels. Thus, the length of an intensity profile was 31 pixels, with one pixel added on the tumor boundary. In addition, each patch was a rectangle with size *w* × *w* in the following experiments, as described in Section 3.4.2. The spacing between sampled patches was both set to 1 pixel in the visual vocabularies construction and in the BoW histograms extraction.

### 4.2. Optimization of System Parameters

#### 4.2.1. Different Local Descriptors

**Figure 8:**
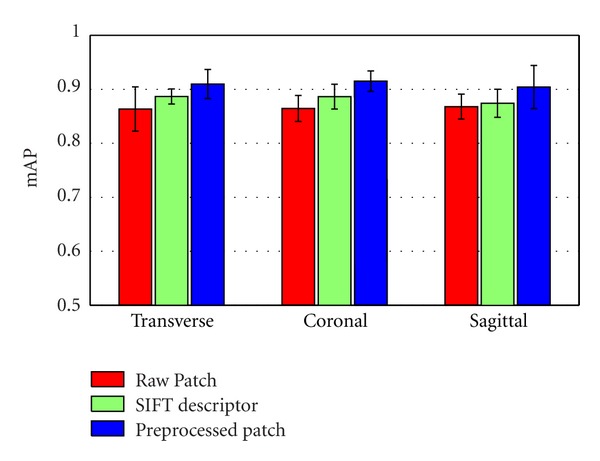
Retrieval performance of the region-specific BoW model with different local descriptors in different views. The bars in the figure show the means and standard deviations of mAP.

In this section, we examined three local descriptors: raw patches, raw patches with normalization and whitening, and SIFT descriptors. We used a 128-dimensional SIFT descriptor implemented by [41] in this task. Moreover, the SIFT descriptors were densely sampled in the tumor region and the margin region, respectively, and followed the flow of the region-specific BoW model construction.  Figure 8 shows the retrieval performance for the region-specific BoW model with different local descriptors in different views. From Figure 8, the mAP of patches with normalization and whitening is higher than that of patches without preprocessing. This result is due to the local contrast enhancement in the preprocessed patch data. Using the patches with normalization and whitening also proved preferable to the SIFT descriptors in the proposed CBIR system.

#### 4.2.2. Different Patch Sizes and Vocabulary Sizes

**Figure 9:**
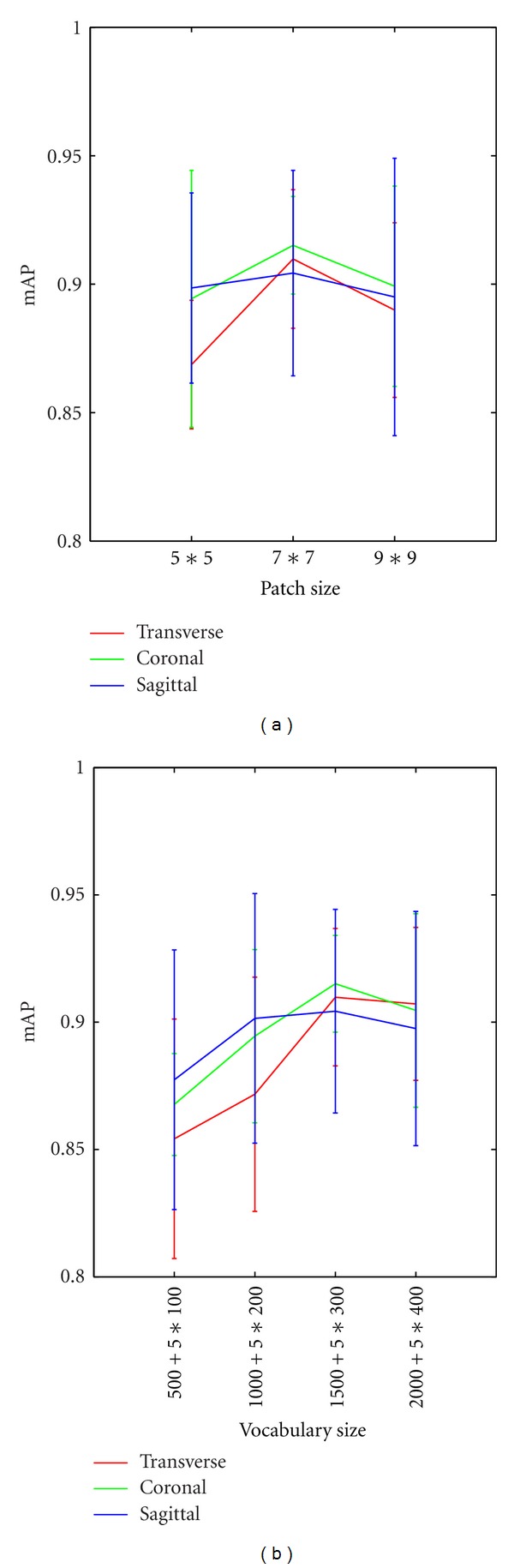
(a) Retrieval performance of the region-specific BoW model with different patch sizes in different views. (b) Retrieval performance of the region-specific BoW model with different vocabulary sizes in different views.

In this task, we assessed the impact of patch size and vocabulary size on the retrieval performance. First, we used three different patch sizes: 5 × 5, 7 × 7, and 9 × 9.  Figure 9(a) depicts the retrieval performance on different patch sizes for the preprocessed patches in the region-specific BoW model, with the distance metric learned by CFML. The bars in Figure 9 show the means and standard deviations of mAP. It can be seen that patch size with 7 × 7 is slightly higher retrieval ability than the other patch sizes. This is because small-sized patches cannot identify objects well, whereas large-sized patches encounter difficulty in finding similar visual words in the vocabulary. Therefore, medium-sized patches can lead to better retrieval performance. Subsequently, to compare the effect of different vocabulary sizes on the retrieval performance, the patch size for preprocessed patches was fixed to 7 × 7 (*w* = 7), and the feature vectors were measured with the distance metric learned by CFML. In Figure 9(b), 5∗*k* represents the dimensionality of the margin region BoW representation, wherein *k* (*k* = 100, 200, 300, and 400) is the vocabulary size in the margin region BoW model. As shown in Figure 9(b), when the vocabulary sizes are 1000 and 300 in the tumor region BoW and margin region BoW, respectively, the mAP achieve the highest scores in different views. Moreover, adding additional visual words after the points mentioned above increases computational time with no significant improvement in the retrieval performance for the region-specific BoW model. Thus, in the following experiments, we used these vocabulary sizes for the region-specific BoW model in different views to balance the computational cost and the performance.

#### 4.2.3. Different Parameters in CFML

**Figure 10:**
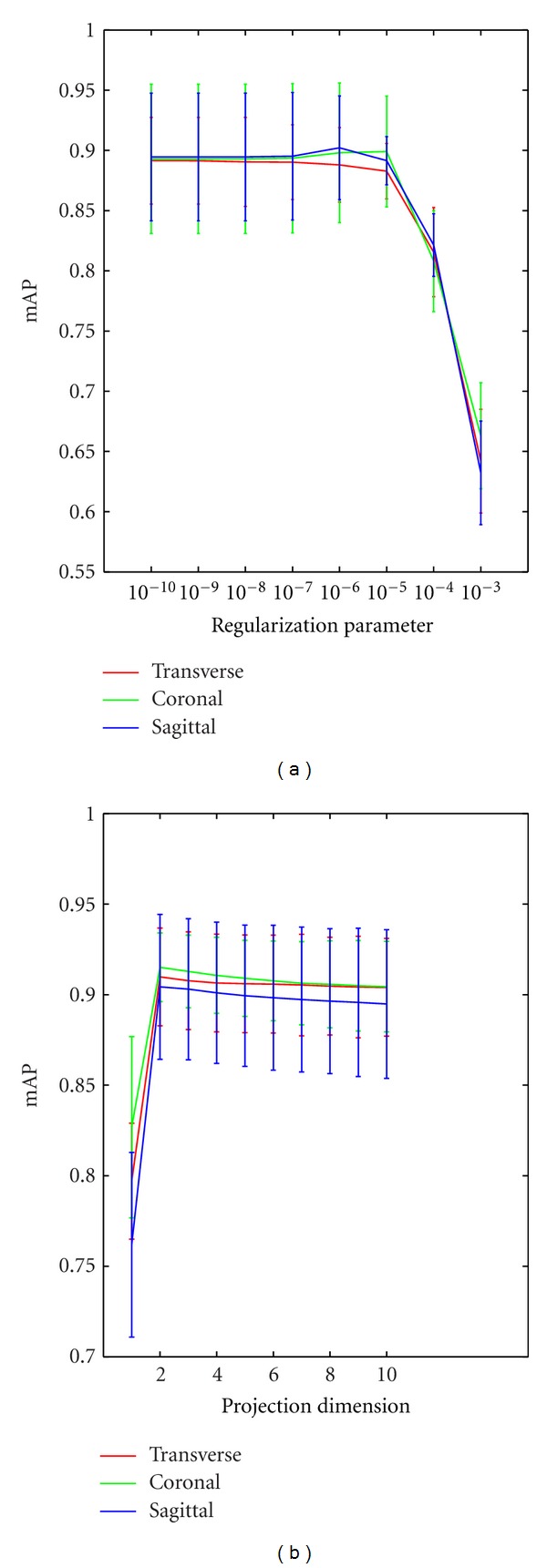
(a) Retrieval performance of the region-specific BoW model with varying regularization parameters in CFML in different views. (b) Retrieval performance of different projection dimensions of the transformation matrix in CFML in different views. The bars in the figure show the means and standard deviations of mAP.

In this task, two parameters in CFML were tuned to obtain the optimal values: the regularization parameter *λ* and the reduced dimensionality of transformation matrix *L*. The optimal local descriptor, patch size and vocabulary sizes were used consistently across all experiments. As described in Section 3.4, the regularization parameter *λ* was used in the training stage to achieve the optimal transformation matrix *L* of CFML. Therefore, we adopted a fivefold cross-validation in each training dataset and ranged *λ* from 10^−10^ to 10^−3^ in this experiment. As depicted in Figure 10(a), varying the value of *λ* until 10^−6^ does not have a significant impact on the performance. Moreover, using a higher value gradually decreases the performance. For this reason, we set the value of *λ* to 10^−6^ in the following experiments. On the other hand, the dimensionality of the transformation matrix *L* can be reduced in the distance metric learned by CFML. The low-dimensional distance metrics can reduce the computational cost and filter noise.  Figure 10(b) shows how varying the projection dimension affects the reliability of the image retrieval. We found that the mAP always gets the highest value with two dimensions in different BoW models and different views. This result is meaningful for the CBIR tasks of speeding up retrieval and reducing storage.

### 4.3. Region-Specific BoW Representation

**Figure 11:**
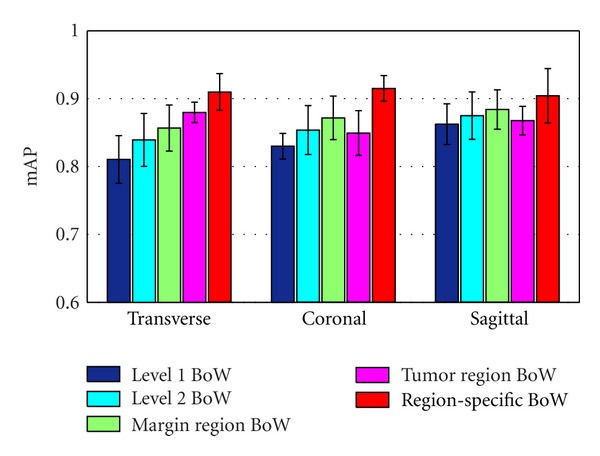
mAP of different BoW representations in different views. The bars in the figure show the means and standard deviations of mAP.

In this experiment, we first used the optimal local descriptor, patch size, and vocabulary sizes in the region-specific BoW model described to construct the level 1 BoW representation, level 2 BoW representation, margin region BoW representation, tumor region BoW representation, and the region-specific BoW representation, respectively. The level 1 BoW representation represents only the single level without incorporating the four subregions in the margin region for constructing BoW representation. The level 2 BoW representation represents only the four subregions without adding the whole margin region for constructing BoW representation (see Figure 4(b)). We then compare the retrieval performance of different BoW representations. As shown in Figure 11, the retrieval performance improved from the level 1 BoW representation to the level 2 BoW representation, which contains finer spatial information. Additionally, levels 1 and 2 together (margin region BoW representation) confers a more significant benefit. Here, we only used two levels to divide the margin region because individual bins yield too few matches when a region is too finely subdivided [29]. Moreover, when the vocabulary size of the margin region is fixed, more subregions emerge which leads to higher dimensionalities of BoW representation. A higher cost is then needed in the following computation of similarity metric. Hence, we make a tradeoff between discriminating power and computational cost in the selection of level numbers in the margin region. Besides, the mAP of the region-specific BoW representation achieves the highest values among different BoW representations in different views. For instance, in the transverse view, the region-specific BoW representation outperforms the tumor region BoW representation and the margin region BoW representation with *t*-test *P* values of 0.0043 and 0.0124, respectively. The gain can be attributed to the additional spatial information achieved in this step.

### 4.4. Retrieval Results with Different Feature Extraction Methods

In this section, we show the comparative results of the category retrieval of brain tumors with different feature extraction methods in the T1-weighted brain CE-MRI dataset. During our experiments, we carefully considered the parameter settings of different methods to achieve fair comparisons. In the rest of this paper, the proposed region-specific BoW representation is based on the optimization procedure mentioned above, and the parameters in CFML are set to the optimal values for retrieval in different methods.

**Figure 12:**
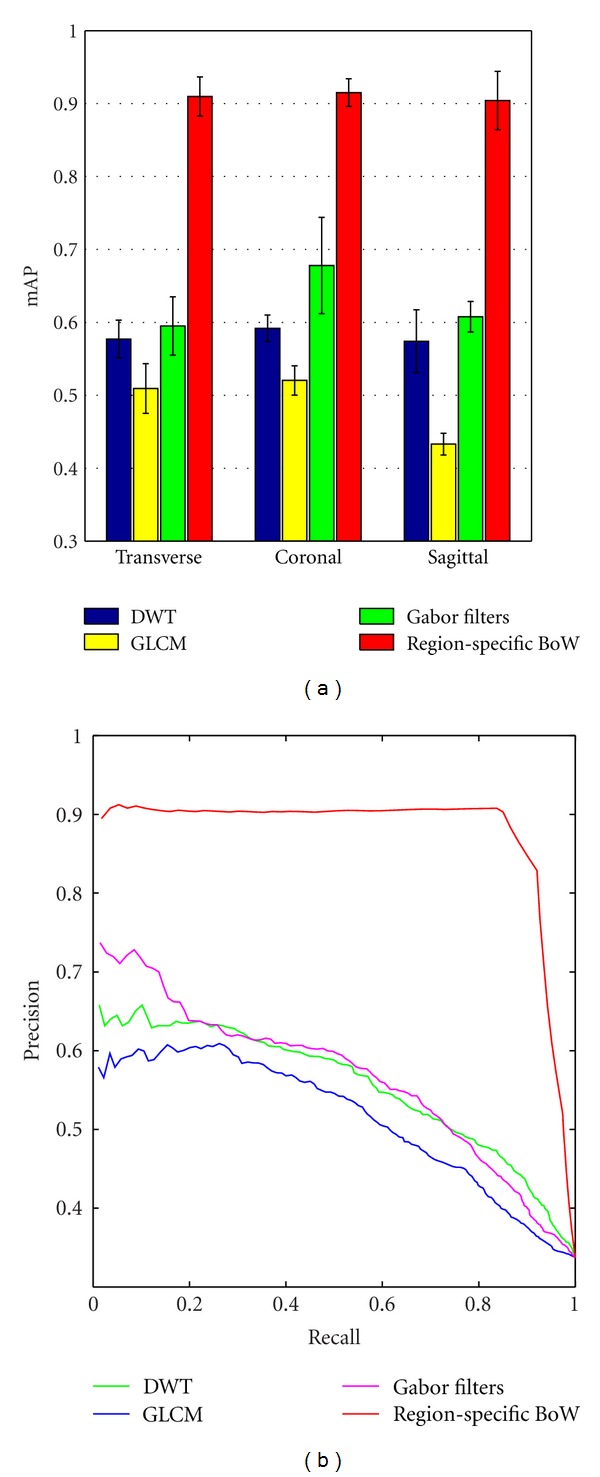
(a) mAP of different texture feature extraction methods in different views. The bars in the figure show the means and standard deviations of mAP. (b) Precision-recall curves of different texture feature extraction methods in the transverse view.

First, comparisons are made between the region-specific BoW model and several traditional texture feature extraction methods, including DWT, GLCM, and Gabor filters. The filter coefficients adopted for computing DWT are the Daubechies wavelets. To calculate DWT, the tumor region on each image was first decomposed into three levels (1 + 3∗3 = 10 subbands) with the wavelet transform. The mean and the variance of the absolute values of the wavelet coefficients in the tumor region corresponding to each sub-band were used to construct a (10∗2 = 20) feature vector. To calculate GLCM, the intensity values in the tumor region were quantized to 32 levels. The interpixel distance was fixed to 1 pixel, and the orientations were set to 0, 45, 90, and 135 degrees to form the GLCMs. Hence, there were four GLCMs with respect to four orientations. Based on the four GLCMs, six statistical parameters (energy, entropy, contrast, variance, correlation, and inverse difference moment) of each GLCM were computed. The mean and the variance of the four values in each of the six parameters were used as the final features (6∗2 = 12). To calculate the Gabor filters, the Gabor wavelet features proposed by Manjunath and Ma [24] were implemented. The number of scales was set to four, and the number of orientations was set to six. The mean and the standard deviations of the magnitude of the transform coefficients in the tumor region were computed as the features, and the feature vector was 48-dimensional (4∗6∗2 = 48).  Figure 12(a) shows a performance comparison in the T1-weighted brain CE-MRI dataset for different texture feature extraction methods. As depicted in Figure 12(a), the mAP of the region-specific BoW model is significantly higher than that of the other texture feature extraction methods in different views (*t*-test *P* value was less than 0.0001). Moreover, Figure 12(b) shows the precision-recall curves of different texture feature extraction methods in the transverse view. The precision-recall curve of the region-specific BoW model is clearly superior to the other methods, which is matching to the highest mAP in Figure 12(a).

**Figure 13:**
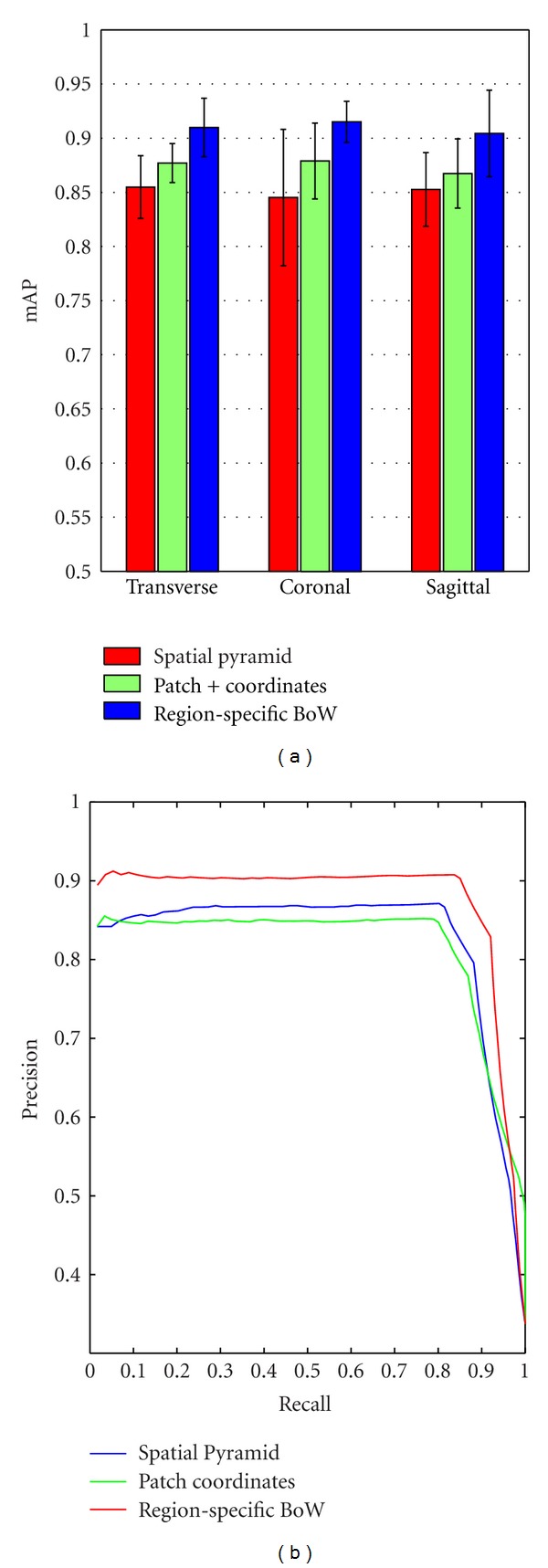
(a) mAP of different spatial BoW methods in different views. The bars in the figure show the means and standard deviations of mAP. (b) Precision-recall curves of different spatial BoW methods in the transverse view.

Next, to validate the performance of the region-specific BoW model, it was compared with two other state-of-the-art approaches, which also added spatial information to the BoW model. First, a spatial pyramid method introduced by [29] was used. For this method, the default parameters suggested by the authors were adopted. The visual vocabulary size was fixed to 200, the pyramid level was set to 3, and the final representation was 4200-dimensional. Second, the method proposed in [30] was applied for this comparison. In this method, patch center coordinates were added to the vector-represented patches to indicate the spatial dependency between patches. For this method, the visual vocabulary sizes in different views were set to 3000. Therefore, the BoW representation of this method was 3000-dimensional, the same value as in the region-specific BoW representation. In addition, the coordinate weight was changed from 1 to 20 and achieved the highest scores with values of 4, 8, and 10 in the transverse, coronal, and sagittal views, respectively. In these two methods, patches with normalization and whitening were densely sampled inside the tumor region as in our proposed method, and the patch size was set to 7 × 7.  Figure 13(a) shows the retrieval performance of different spatial BoW methods in different views. From Figure 13(a), the mAP of the patch combining coordinates method is not significantly higher than that of the spatial pyramid method with *t*-test *P* values of 0.3598, 0.1431, and 0.3792 in the transverse, coronal, and sagittal views, respectively. This result may be due to the difficulty of catching statistical information when the locations of the tumors belonging to the same type significantly vary in different brain images. Therefore, the patch combining coordinates method is less discriminative when the aforementioned case happens. On the other hand, our region-specific BoW model achieves the highest values among the three spatial BoW methods in different views (*t*-test *P* value was less than 0.0484). As shown in Figure 13(b), the precision of the region-specific BoW model is superior to the other two methods until the recall reaches the value of 0.9.

### 4.5. Retrieval Examples

Retrieval performance of the region-specific BoW model for the different categories of brain tumors was evaluated. The results are listed in Table 2. In Table 2, the values represent the mean and the standard deviation of mAP, Prec@10, and Prec@20, respectively, for five runs. From Table 2, mAP, Prec*@*10, and Prec*@*20 for glioma are higher than those for the other two tumor types, supporting that the region-specific BoW representations contain discriminative information to distinguish gliomas from other tumor types (*t*-test *P* value was less than 0.0269). At the same time, the retrieval performance of meningiomas and pituitary tumors is similar (*t*-test *P* value was more than 0.093). The inferior performance of meningiomas and pituitary tumors is likely due to their similar appearance and the unbalanced distribution of samples in the dataset.

**Figure 14:**
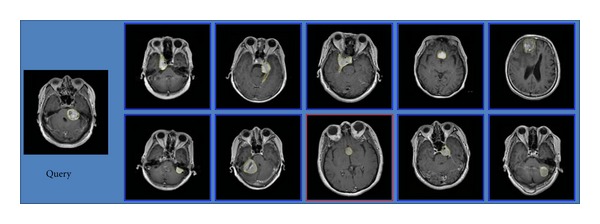
A query of meningioma and its top 10 retrieved results. The irrelevant tumor in this figure is a pituitary tumor.

**Figure 15:**
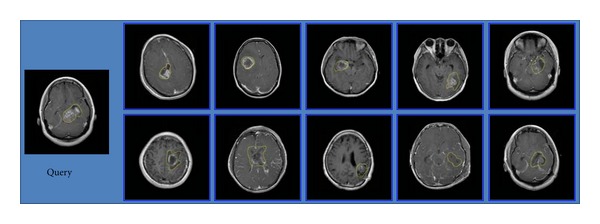
A query of glioma and its top 10 retrieved results.

**Figure 16:**
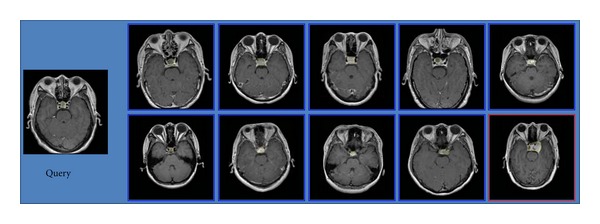
A query of pituitary tumor and its top 10 retrieved results. The irrelevant tumor in this figure is a meningioma.

Three examples of retrieval performance for the different categories of brain tumors with the region-specific BoW representation in the transverse view are shown in Figures 14, 15, and 16, respectively. Among these figures, the leftmost is a query image. The blue frame indicates that the images are relevant to the query image; conversely, the red frame indicates that the images are irrelevant to the query image. There is an irrelevant image among the top 10 retrieved images in Figures 14 and 16, respectively. All the top 10 images for the query of glioma are relevant in Figure 15. These results conform to the lower Prec*@*10 of meningiomas and pituitary tumors and to higher Prec*@*10 of gliomas in Table 2.

## 5. Discussion and Conclusion

In this paper, we presented a region-specific BoW method for the retrieval of medical images with lesions. The region-specific BoW model, which adds spatial information to the BoW model, was proposed to capture the statistical information of the intensity values in the lesion region and the intensity variation of the lesion-surrounding region. In addition, we provided a comprehensive overview of the methodology and its retrieval application to brain tumors in a T1-weighted CE-MRI dataset. We also investigated the effects of various parameters on the overall retrieval and tuned the system to achieve a high score in the retrieval of brain tumors in a T1-weighted CE-MRI dataset.

Two key characteristics that were evaluated throughout this paper are the patches with preprocessing and the use of spatial information as part of the BoW representation. As shown in Figure 8, the retrieval performance of patches with normalization and whitening is better than the performance of those without preprocessing due to the uncorrelated components and the local contrast enhancement within a preprocessed patch data. On the other hand, an obvious advantage of using preprocessed patches over SIFT descriptors for the brain tumor CE-MRI dataset is shown in Figure 8. Since SIFT descriptors can well describe the variety of the structure and the tissues in brain tumors lack structural information, these descriptors are insufficient to extract the distinctive information of the brain tumors in MRI images. Moreover, these results lend further credence to our earlier suggestion that intensity value is a powerful tool in the retrieval application for brain tumors in MRI images. As it incorporates the spatial information and domain knowledge to the BoW model, the region-specific BoW representation is advantageous in most scenarios. A significant improvement was shown in Figure 11, when the tumor region BoW and the margin region BoW were combined for the retrieval performance. As mentioned in Section 2, the BoW model is useful in the application of the CBIR system. Indeed, in our experiments, the proposed region-specific BoW model achieved a mean mAP of 91.0% in different views, which was significantly higher than the other commonly used texture feature extraction methods (Figure 12). This result also supports the suitability of the BoW framework in learning the task-specific and subtle representation in MRI images. The region-specific BoW representation was also compared with two spatial BoW methods, and a better performance was achieved as shown in Figure 13.

The preliminary results demonstrate that the region-specific BoW model can achieve the mAP of 91.0%, 91.5%, and 90.4% in the transverse, coronal, and sagittal views, respectively. In addition, among the three tumor types, the retrieval of glioma performs best and achieves 95.3%, 93.4%, and 93.4% for the mean of mAP, Prec@10, and Prec@20 in different views, respectively. Overall, these results suggest that it is feasible to separate the tumor images into tumor regions and margin regions and combine this spatial information with the BoW model to retrieve similar lesions in the brain CE-MRI images.

In the developed retrieval system, the brain tumors in MRI images are outlined manually, which is not time efficient and convenient. An optional solution is to adopt an automatic or interactive segmentation of brain tumors. The accuracy of this method may be lower than that of the manual segmentation by experts. However, the margin region used in this paper can compensate for this drawback. The intensity profiles are extracted along the tumor boundary normal that contains pixels inside and outside the tumor boundary. As long as the contour of the segmentation is near the real tumor boundary, the intensity variety around the tumor can still be caught by the margin region. Another competitive and practical solution for this problem is to develop an automatic detection method for tumor regions, a solution we intend to focus on in our future work.

## Figures and Tables

**Table 1:** Number of different brain tumors in different views.

View	Tumor type	Number of patients	Total
Transverse	Meningiomas	58	190
	Gliomas	72	
	Pituitary tumors	60	

Coronal	Meningiomas	57	190
	Gliomas	72	
	Pituitary tumors	61	

Sagittal	Meningiomas	77	215
	Gliomas	76	
	Pituitary tumors	62	

**Table 2:** Retrieval performance of the region-specific BoW model for the different categories in different views.

View	Tumor category	mAP	Prec@10	Prec@20
Transverse	Meningioma	92.0 ± 5.8	89.6 ± 7.5	89.2 ± 7.6
	Glioma	95.0 ± 3.3	93.1 ± 4.7	93.1 ± 4.7
	Pituitary tumor	87.6 ± 4.6	82.5 ± 6.6	82.7 ± 6.8

Coronal	Meningioma	87.6 ± 5.0	84.0 ± 8.3	83.7 ± 8.8
	Glioma	95.3 ± 2.9	93.3 ± 4.4	93.3 ± 4.4
	Pituitary tumor	89.5 ± 2.5	86.8 ± 2.3	86.8 ± 2.9

Sagittal	Meningioma	91.2 ± 4.0	87.8 ± 8.0	87.7 ± 8.0
	Glioma	95.6 ± 2.9	93.7 ± 4.0	93.7 ± 4.0
	Pituitary tumor	82.1 ± 1.0	76.7 ± 1.3	76.6 ± 1.3
